# Music improves verbal memory encoding while decreasing prefrontal cortex activity: an fNIRS study

**DOI:** 10.3389/fnhum.2013.00779

**Published:** 2013-11-22

**Authors:** Laura Ferreri, Jean-Julien Aucouturier, Makii Muthalib, Emmanuel Bigand, Aurelia Bugaiska

**Affiliations:** ^1^Laboratory for the Study of Learning and Development, CNRS UMR 5022, Department of Psychology, University of BurgundyDijon, France; ^2^Institut de Recherche et Coordination Acoustique/Musique, STMS CNRS UMR9912Paris, France; ^3^Movement to Health, EUROMOV, Montpellier-1 UniversityMontpellier, France

**Keywords:** music, verbal memory, encoding, prefrontal cortex, fNIRS

## Abstract

Listening to music engages the whole brain, thus stimulating cognitive performance in a range of non-purely musical activities such as language and memory tasks. This article addresses an ongoing debate on the link between music and memory for words. While evidence on healthy and clinical populations suggests that music listening can improve verbal memory in a variety of situations, it is still unclear what specific memory process is affected and how. This study was designed to explore the hypothesis that music specifically benefits the encoding part of verbal memory tasks, by providing a richer context for encoding and therefore less demand on the dorsolateral prefrontal cortex (DLPFC). Twenty-two healthy young adults were subjected to functional near-infrared spectroscopy (fNIRS) imaging of their bilateral DLPFC while encoding words in the presence of either a music or a silent background. Behavioral data confirmed the facilitating effect of music background during encoding on subsequent item recognition. fNIRS results revealed significantly greater activation of the left hemisphere during encoding (in line with the HERA model of memory lateralization) and a sustained, bilateral decrease of activity in the DLPFC in the music condition compared to silence. These findings suggest that music modulates the role played by the DLPFC during verbal encoding, and open perspectives for applications to clinical populations with prefrontal impairments, such as elderly adults or Alzheimer’s patients.

## INTRODUCTION

Listening to music engages the whole brain through a diverse set of perceptive and cognitive operations, and equally diverse neural substrates (Altenmüller, 2003). As most of these neural substrates also intervene in other activities, it is increasingly believed that music can benefit non-musical abilities, and most notably language (Abbott and Avins, 2006; Patel, 2011). In particular, there is an ongoing debate in the field of music and cognitive stimulation on whether music can be used to enhance verbal memory. On the one hand, music is a complex auditory stimulus which evolves through time and which has a strong emotional impact (Blood and Zatorre, 2001; Salimpoor et al., 2013). As such, music can provide considerable additional cues which are likely to enrich the encoding of an event. On the other hand, musical information was also claimed to negatively affect memory by attracting participants’ attention away from the information to be remembered, generating a dual task situation with poorer memory performance than in a silent situation (Racette and Peretz, 2007; Moussard et al., 2012). In the last 20 years, several studies were conducted in order to understand when and how music can have a positive effect on memory. Research on western music indicates that musical training (Chan et al., 1998; Ho et al., 2003; Franklin et al., 2008) and also simple exposure to music leads to benefits on short- and long-term verbal memory in healthy and clinical populations (Balch et al., 1992; Balch and Lewis, 1996). In 1994, Wallace showed that text is better recalled when heard as a song rather than speech, suggesting that musical context can assist in learning and retrieving words. In clinical settings, short (i.e., music played as a background in a memory task) and long-lasting (i.e., in a music-therapy program) auditory stimulations with music were both shown to improve category fluency in a verbal fluency task in both healthy elderly and Alzheimer’s patients (Thompson et al., 2005), speech content and fluency in patients with dementia (Brotons and Koger, 2000), and verbal memory in stroke patients (Särkämö et al., 2008). Additionally, verbal material is more efficiently retrieved when sung than spoken in multiple sclerosis (Thaut et al., 2005), aphasics (Racette et al., 2006), and Alzheimer’s patients (Simmons-Stern et al., 2010).

Such evidence suggests that music provides contextual cues that contribute to episodic memory processes. Episodic memory (Tulving, 1972) enables conscious recollection of personal experiences and events from the past (Wheeler et al., 1997). Encoding is a crucial aspect of episodic memory and it is tightly related to the contribution of contextual factors such as location, time, prevailing conditions, and converging multisensory and emotional stimuli (Eich, 1985; Hamann, 2001; Kensinger and Corkin, 2003; Hupbach et al., 2008). Neuroimaging and behavioral data have clearly shown that the capacity to retrieve correct information depends on its successful encoding (e.g., Prince et al., 2005; Hertzog et al., 2010). Furthermore, richer contexts enhance the encoding of contextual information associated to an item and can be subsequently used as mnemonic cues during retrieval, facilitating the access to the target item (Lövdén et al., 2002; Kroneisen and Erdfelder, 2011). It has been shown that enriching the context of encoding through, e.g., enacted encoding (Lövdén et al., 2002) or with emotional valence stimuli (see Hamann, 2001 for a review) can enhance memory performance at retrieval. It is therefore possible that the greatest value of music for memory is to provide mnemonic processes with a particularly rich and helpful context during the encoding phase of episodic memory.

Memory encoding and retrieval processes are supported by a broad brain network that involves the medio-temporal and posterior parietal areas, the hippocampus, and the prefrontal cortex (PFC), the latter being particularly important for episodic memory (Spaniol et al., 2009; Manenti et al., 2012). Different sub-regions of the PFC are recruited by different mnemonic processes: according to the hemispheric encoding/retrieval asymmetry (HERA) model (Tulving et al., 1994), left PFC activation is greater for encoding than retrieval, while right PFC activation is greater for retrieval than encoding. Although the PFC sub-region most constantly associated with memory in neuroimaging studies is the ventrolateral prefrontal cortex (VLPFC – BA 44-45-47), the dorsolateral-prefrontal cortex (DLPFC – BA 9 and 46) has recently gained importance for the specific investigation of memory encoding processes. In particular, it has been shown that the DLPFC, mainly in the left hemisphere, plays a crucial role for organizational, associative (Murray and Ranganath, 2007; Ranganath, 2010) and semantic (Innocenti et al., 2010) memory encoding. As discussed by Blumenfeld and Ranganath (2007), DLPFC activation seems to be more specifically sensitive to demands for organizational processing and it may support long term memory by building associations among items that are active in memory.

Functional near-infrared spectroscopy (fNIRS) is an optical neuroimaging technique that can non-invasively monitor cortical tissue oxygenation (oxygenated-O_2_Hb and deoxygenated-HHb hemoglobin concentration changes) during cognitive, motor, and sensory stimulation (Jobsis, 1977; Ferrari and Quaresima, 2012). In the last 20 years, the use of fNIRS in cognitive neuroscience has constantly increased (Ferrari and Quaresima, 2012). In the field of memory research in particular, fNIRS studies have revealed an increase in PFC oxygenation patterns (i.e., an increase in O_2_Hb and concomitant decrease in HHb concentrations) during working memory and attention tasks in healthy and clinical populations (see Cutini et al., 2012 for a review). However, the current literature only has a limited number of fNIRS studies (Kubota et al., 2006; Matsui et al., 2007; Okamoto et al., 2011) investigating episodic encoding-retrieval processes. In the field of music cognition, a few fNIRS studies were recently conducted in order to investigate the emotional response to music (Moghimi et al., 2012a; Moghimi S et al., 2012b). However, no fNIRS study has yet looked at a possible role of music in memory encoding.

The previous fNIRS studies that have documented facilitating factors on memory encoding, e.g., strategies to memorize words (Matsui et al., 2007), or pharmacological stimulants such as methylphenidate (Ramasubbu et al., 2012), have repeatedly shown that such factors deactivate, rather than more greatly activate, regions of the PFC – as if they were “less-demanding” (Matsuda and Hiraki, 2004, 2006). Similar reductions of PFC activation were shown using fMRI when there were strong semantic associations between words (Addis and McAndrews, 2006). These evidence suggest that facilitatory cues (e.g., strategies, pharmacological stimulant, strong semantic associations) during verbal encoding could result in less involvement of high cognitive functions mediated by PFC regions (such as DLPFC) known to be usually crucial during memory encoding processes.

The present study addresses the music and memory debate using a source memory paradigm: participants were asked to memorize both lists of words and the context/source (either music or silence) in which words were encoded. The critical new point of the present study was to assess whether the presence of background music during the encoding of verbal material results in different memory-specific cortical patterns of activations than episodic encoding in silence. We used fNIRS to monitor the DLPFC bilaterally during the encoding of verbal material with or without background music context. Our hypothesis is that music may enhance verbal encoding by providing a helpful context which can facilitate organizational, associative, and semantic processes. If so, such an effect of music on memory encoding processes should be linked to both behavioral performance and PFC activity. More specifically, we consider a facilitatory effect of music during verbal encoding should result in a better recognition performance and deactivation of DLPFC activity during the music encoding condition compared to the silent condition.

## MATERIALS AND METHODS

### PARTICIPANTS

Twenty-two young healthy students at University of Burgundy (11 female, mean age 23.5 ± 4.3 years) took part in the experiment in exchange of course credits. All the participants were right-handed, non-musicians, French-native speakers, and reported having normal or corrected-to-normal vision. None were taking medication known to affect the central nervous system. Informed written consent was obtained from all participants prior to taking part in the experiment. The study was anonymous and fully obeyed to the Helsinki Declaration, Convention of the Council of Europe on Human Rights and Biomedicine.

### EXPERIMENTAL PROCEDURE

Subjects were seated in a chair in front of a computer in a quiet, dim room. Each participant was subjected to a memory encoding task while their PFC activation was monitored using fNIRS neuroimaging and then behaviorally tested in a retrieval task. After the eight fNIRS probe-set was adjusted on the forehead scalp overlaying the DLPFC (see fNIRS section below for a description) and the in-ear headphones inserted, subjects were informed that they would be presented with different lists of words with two different auditory contexts: music or silence. They were asked to memorize both the lists of words and the context in which words were encoded.

Verbal stimuli consisted of 42 taxonomically unrelated concrete nouns selected from the French “Lexique” database (New et al., 2004, ). They were randomly divided into 6 lists of words (7 words each list, 21 words for each encoding condition), equated for word length and occurrence frequency. In the music encoding condition, the background music used in all blocks was an upbeat, acoustic jazz piece (“If you see my mother” by Sidney Bechet), chosen for its positive valence and medium arousal quality.

**FIGURE 1:**
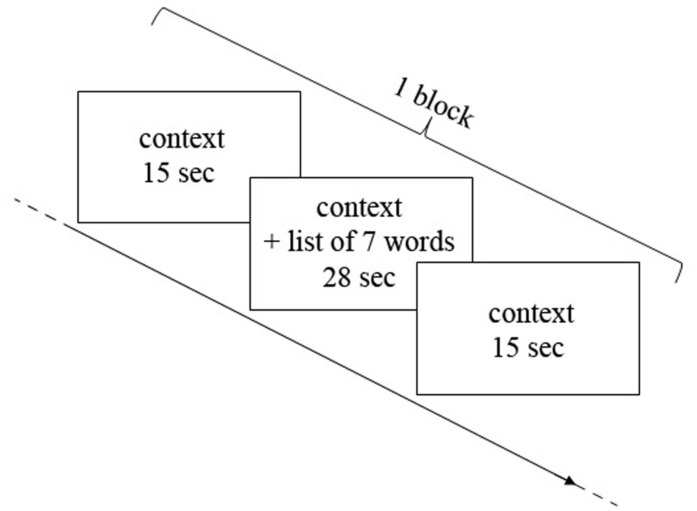
**Representation of one block of encoding.** Each block consisted in 15 s of context alone (music or silence in the earphones), then 28 s of context and words encoding (seven words for each block, 4 s for each word) and then again 15 s of context alone.

The encoding phase consisted of three blocks of “music encoding” and three blocks of “silence encoding” intermixed with 30-s rest periods. In each block, seven words were displayed successively in the presence of a music or silence auditory context. The audio stimulation started 15 s before the first word was displayed, continued during the sequential display of words, and ended 15 s after the last word. Words displayed in each block were paced at 4 s per word, amounting to 28 s for the sequential presentation of seven words. Each block therefore had a duration of 58 s (15 s context, 28 s words, 15 s context), and was followed by a 30 s rest (silent) between each block (**Figure 1**). The order of music/silence blocks was counterbalanced, as well as the order of word lists and the order of words in the lists. During the rest periods subjects were instructed to try to relax and not to think about the task any longer; in contrast, during the context-only phases of the blocks (i.e., silence and music blocks), participants were instructed to concentrate on a fixation cross on the screen and to prepare/focus on the task. The entire encoding phase, together with fNIRS recording, took about 10 min.

Prior to the retrieval phase, subjects performed two 5-min interference tasks: a “X-O” letter-comparison task (Salthouse et al., 1997) and a “plus–minus” task (Jersild, 1927; Spector and Biederman, 1976). Subjects were then tested for item and source memory recognition. We used item-memory and source-memory tasks (Glisky et al., 1995) in order to evaluate the subjects’ memory for the context of encoding. The retrieval test included the 42 words presented previously, together with 42 new words which were lure items matched for word length and occurrence frequency. For each word, subjects were asked to judge if they had already seen the word before (yes/no button on the keyboard; item-memory task). If they believed they had, they were asked to indicate in which context they saw the word (music/silence/I don’t know; source-memory task). The presentation of task instructions and stimuli as well as the recording of behavioral responses were controlled by the E-Prime software (Psychology Software Tools, Inc.) running on a laptop with a 15^′′^ monitor.

### FNIRS MEASUREMENTS

**FIGURE 2:**
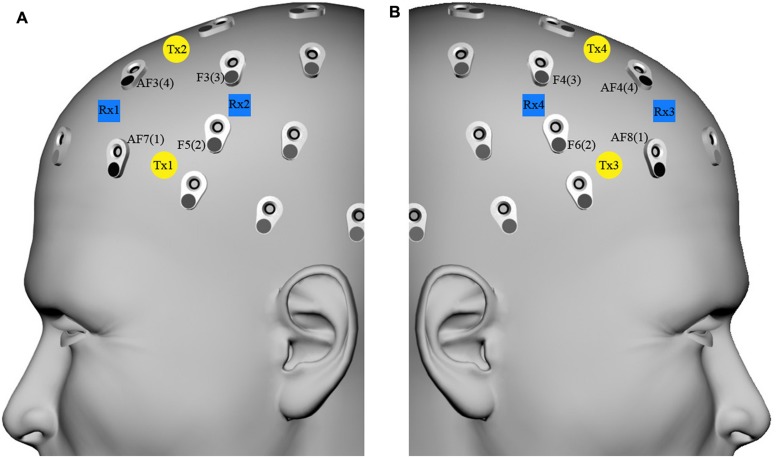
**fNIRS optode localization on the forehead scalp region overlying the dorsal part of the prefrontal cortex.** fNIRS transmitters [**(A)** Tx1–Tx2, **(B)** Tx3–Tx4, yellow circles) and receivers [**(A)** Rx1–Rx2, **(B)** Rx3–Rx4, blue squares) were placed on the **(A)** and **(B)** forehead scalp region, which corresponded to AF7/8, F5/6, F3/4, and AF3/4 EEG channels (international 10/10 system), respectively renamed left/right channels 1, 2, 3, and 4.

An eight-channel fNIRS system (Oxymon MkIII, Artinis Medical Systems B.V., The Netherlands) was used to measure the concentration changes of O_2_Hb and HHb (expressed in micromoles) using an age-dependent constant differential path-length factor given by 4.99 + 0.0067*(age 0.814; Duncan et al., 1996). Data was acquired at a sampling frequency of 10 Hz. The eight fNIRS optodes (four emitters and four detectors) were placed symmetrically over the dorsal part of the PFC (Brodmann Areas 46 and 9, EEG electrodes AF7/8, F5/6, F3/4, and AF3/4 of the international 10/10 system; Okamoto et al., 2004; Jurcak et al., 2007), and the distance between each emitter and detector was fixed to 3.5 cm (**Figure 2**).

To optimize signal-to-noise ratio during the fNIRS recording, the eight optodes were masked from ambient light by a black plastic cap that was kept in contact with the scalp with elastic straps, and all cables were suspended from the ceiling to minimize movement artifacts (Cui et al., 2011). During data collection, O_2_Hb and HHb concentration changes were displayed in real time, and the signal quality and the absence of movement artifacts were verified.

### DATA AND STATISTICAL ANALYSIS

#### Behavioral data

Each subject’s item- and source-memory accuracy (hit) rates (number of hits for each condition during the yes/no recognition) as well as false alarms were calculated for both the silence and music conditions. To examine source memory, we analyzed the proportion of correct source judgments among item-memory hits. A paired *t*-test was used to compare the item- and source- memory scores between the silence and music conditions. One sample *t*-tests were used to ascertain that all the scores were significantly above chance.

#### fNIRS data

For each of the eight fNIRS measurement points, the O_2_Hb and HHb signals were first low-pass filtered to eliminate task-irrelevant systemic physiological oscillations (fifth order digital Butterworth filter with cutoff frequency 0.1 Hz).

**FIGURE 3:**
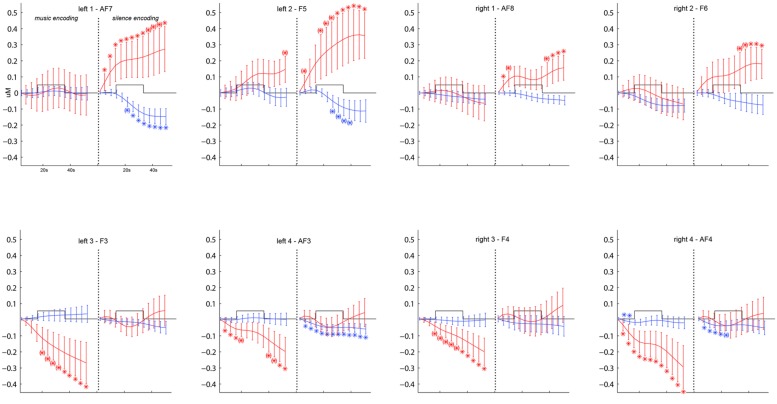
**Grand-average (±SEM) time course of prefrontal cortex O_**2**_Hb (red lines) and HHb (blue lines) concentration changes (vertical axis) over the left and right hemisphere during memory encoding (horizontal axis: time) for the silence (right side) and music (left side) conditions.** * and (*) show, respectively, significant (*p* < 0.05) and marginally significant (0.05 < *p* < 0.09) time points compared to baseline obtained by *post hoc* Fisher’s LSD comparisons.

In order to ascertain the DLPFC activation during the word encoding task as compared to the rest phase, we first ran a complete timecourse analysis on the O_2_Hb and HHb signals using a 2(music/silence condition) × 2(left/right hemisphere) × 4(optodes) × 13(successive measures of concentrations, averaged over 5 s windows with the last 10 s of the rest phase as baseline) repeated-measures ANOVA, on which Fisher’s LSD *post hoc* comparisons determine which steps of the O_2_Hb and HHb time course showed significant increase/decrease of O_2_Hb and HHb as compared to the baseline point set during the rest phase (**Figure 3**).

In order to determine the amount of activation during the encoding phase for the two conditions, data in each of the six experimental blocks was baseline corrected using the mean of the O_2_Hb and HHb signals during the first 5 s of each block (i.e., during context-only phase, before the start of word encoding). We then sample-to-sample averaged (i.e., 10 samples/s) the baseline-corrected signals over the three blocks of each condition, yielding one average music and silence O_2_Hb and HHb signal per participant. We then computed the maximum O_2_Hb (max- O_2_Hb) value and the minimum HHb (min-HHb) value over the 28 s stimulus window (i.e., from *t* = 15 s to *t* = 42 s), for both the music and silence average block of each participant. The peak concentrations (max-O_2_Hb, min-HHb) were analyzed using a repeated measure MANOVA with 2(music/silence condition) × 2(left/right hemisphere) repeated factors and optodes (4) as a multivariate. The significance level was set at *p* < 0.05.

## RESULTS

### BEHAVIORAL RESULTS

Both the item and source memory scores were significantly above chance (one sample *t*-test, *p* < 0.003), demonstrating that participants did not encounter strong difficulties to remember the specific context in which words were presented.

There was a statistically significant difference in item recognition performance between the music (mean**= 18.36, SD**= 2.84) and silence [mean**= 16.59, SD**= 3.98; *t*(21) = 2.63, *p* = 0.016] conditions, with improved recognition in the musical condition. However, there was no significant difference (*p* > 0.05) in source memory performance between music (0.67 ± 0.22) and silence (0.68 ± 0.22) conditions.

### fNIRS RESULTS

**Figure 3** shows the grand average time course of PFC O_2_Hb and HHb concentration changes at each of the eight fNIRS channels in the music and silence encoding conditions. The repeated-measure ANOVA on the O_2_Hb and HHb timecourse series revealed a main effect of condition (*F* = 8.130, *p* = 0.01), corresponding to significantly greater O_2_Hb increases bilaterally in the silence than music condition. Although the increases in O_2_Hb are visible bilaterally during the silence condition (especially in bilateral channels 1 and 2 as shown by *post hoc* LSD Fisher comparisons), together with a decrease in HHb (in particular for left channels 1, 2 and right channel 2), the music condition was associated with a strong bilateral decrease of O_2_Hb (underlined by significant LSD Fisher *post hoc* comparisons especially for bilateral channels 3 and 4).

**FIGURE 4:**
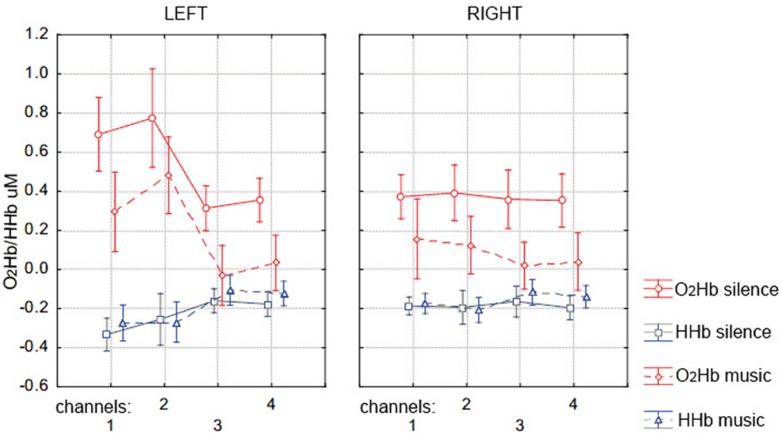
**Mean (±SD) of the prefrontal cortex max-O_2_Hb (red lines) and min-HHb (blue lines) concentration values over the left (channels 1, 2, 3, and 4, corresponding to EEG channels AF7, F5, AF3, F3) and right (channels 1, 2, 3, and 4, corresponding to EEG channels AF8, F6, F4, AF4) hemisphere during memory encoding for the silence (solid lines) and music (dotted lines) condition**.

**Figure 4** shows the group mean of max-O_2_Hb and min-HHb values recorded at each of the eight fNIRS channels on the PFC for the silence and music conditions. The repeated-measure MANOVA on max-O_2_Hb values revealed a statistically significant main effect of condition [*F*(4,18) = 4.207, *p* = 0.008], with greater O_2_Hb increases bilaterally in the silence than music condition, and a significant main effect of channel laterality [*F*(4,18) = 5.006, *p* = 0.003], with greater O_2_Hb increases in the left hemisphere regardless of condition. Although there was no effect of condition for min-HHb values, which is typical with several other NIRS studies (e.g., Matsui et al., 2007; Okamoto et al., 2011), there was a significant effect of laterality [*F*(4,18) = 3.783, *p* = 0.013], with greater values in the right hemisphere (which is coherent with smaller O_2_Hb values, i.e., overall smaller O_2_Hb increases in the right hemisphere).

## DISCUSSION

The present study shows that a background musical context during the encoding of verbal material modulates the activation of the DLPFC and, at the same time, facilitates the retrieval of the encoded material. Despite a few recent studies (e.g., Racette and Peretz, 2007; Moussard et al., 2012) reporting a perturbing effect of music on the memorization of verbal material, a consistent part of the literature (e.g., Balch et al., 1992; Wallace, 1994; Balch and Lewis, 1996; Chan et al., 1998; Brotons and Koger, 2000; Ho et al., 2003; Thaut et al., 2005; Thompson et al., 2005; Racette et al., 2006; Franklin et al., 2008; Särkämö et al., 2008; Simmons-Stern et al., 2010) claims that music can have a positive effect on memory in both healthy and clinical populations. However, most of these studies had remained on a behavioral level. The critical new point of the present study was to also track the brain activation response during the encoding phase with fNIRS: we found that improved word recognition coincides with reduced DLPFC activation in musical encoding compared to silence encoding.

### BEHAVIORAL RESULTS

Our behavioral results showed an effect of music on subsequent item recognition memory performance, although this did not extend to a source memory performance. We found that music played during encoding facilitates item recognition. The role of background music in learning and memory tasks is still an open and debated question in the literature (e.g., Schellenberg, 2003, 2005; De Groot, 2006; Peterson and Thaut, 2007; Jäncke and Sandmann, 2010). Research in music cognition is increasingly aware of the fact that it is necessary, rather than stating a general and reliable positive effect of music, to disentangle which experimental paradigm can lead to memory improvements through music, when and with whom. Our results are in line with previous studies on healthy subjects (Balch et al., 1992; Wallace, 1994; Balch and Lewis, 1996; De Groot, 2006) and clinical populations (Brotons and Koger, 2000; Ho et al., 2003; Thaut et al., 2005; Thompson et al., 2005; Racette et al., 2006; Franklin et al., 2008; Särkämö et al., 2008; Simmons-Stern et al., 2010) which showed a positive role of music in verbal memory encoding. Consistent with the view that context is crucial during episodic memory encoding, our findings support the idea that music provides rich and helpful contextual cues that are useful for subsequent item recognition.

### fNIRS RESULTS

The novel finding of the study was that DLPFC activation was significantly higher during the silence than music encoding condition (Figures 3 and 4). As predicted in our hypothesis, the facilitatory effect of music during verbal encoding resulted not only in better recognition performance, but also deactivation of DLPFC activity. On the one hand, encoding PFC activity in the silent condition followed the classical hemodynamic response to neuronal activation, showing a bilateral increase of O_2_Hb together with a decrease of HHb as compared to baseline. This result confirms the involvement of the DLPFC in episodic memory encoding (Blumenfeld and Ranganath, 2007; Murray and Ranganath, 2007; Innocenti et al., 2010). On the other hand, encoding in the music condition showed a bilateral reversed PFC hemodynamic response (with a sustained decrease in O_2_Hb and minimal change in HHb), which only returned to baseline at the end of each music block (see **Figure 3**). This result suggests that the DLPFC was deactivated during word encoding in the musical context and that music can strongly modulate the activity of the bilateral DLPFC. Similar PFC deactivation have already been shown by fNIRS studies investigating human cognition (Matsuda and Hiraki, 2006, 2004), and specifically in verbal learning tasks when subjects were helped to memorize words by a given strategy (Matsui et al., 2007) or by a pharmacological stimulant (Ramasubbu et al., 2012).

However, to the best of our knowledge, none of the previous fNIRS studies which investigated memory processes or music perception reported music-specific PFC deactivation. In the present study, this PFC deactivation during memory encoding with a musical context could be the manifestation of our hypothesis that music plays a facilitating, less-demanding role for the PFC during word encoding.

fNIRS analysis of the maximum O_2_Hb concentration values reached during word encoding in the music and silence conditions revealed also a significant main effect of lateralization. As predicted, we found greater PFC activation (represented by O_2_Hb increases) in the left than the right hemisphere during the entire encoding phase (especially for channels 1 and 2, see **Figure 4**). This result is in line with the hemispheric left prefrontal asymmetry during the encoding of verbal material, as predicted by the HERA model (Tulving et al., 1994; Nyberg et al., 1996), and confirms the feasibility of fNIRS neuroimaging for the study of long-term memory processes (Kubota et al., 2006; Matsui et al., 2007; Okamoto et al., 2011).

It is important to discuss the possible mechanisms by which music may act on the PFC during memory encoding tasks. The PFC, specifically the DLPFC, is known to be recruited during tasks demanding organizational (Blumenfeld and Ranganath, 2007) and relational inter-item processing during encoding (Murray and Ranganath, 2007). Therefore, one possible interpretation of the deactivation of the PFC (i.e., O_2_Hb decrease) during music in the present study is that music helps to generate inter-item and item-source relationships, without demanding high-cognitive PFC processes. Investigating the correlation between PFC activity and semantic associations during word encoding with fMRI, Addis and McAndrews (2006) found that greater semantic associations correlated with reduced activity in the inferior frontal gyrus (IFG) region of the PFC. A recent fNIRS study by Ramasubbu et al. (2012) also supports this explanation. The authors gave methylphenidate (a central nervous system stimulant) or placebo to subjects and measured PFC activation during a working memory task (N-back). They found a reduction in PFC O_2_Hb from baseline together with better behavioral performance, which the authors suggested was due to methylphenidate improving neuronal efficiency or signal–noise ratio during the memory task. In the present study, the decreased PFC activity observed during the music condition could therefore indicate better neuronal efficiency.

The musical context may afford efficient mnemonic strategies to bind items between one another, and/or to bind items to music, so that less PFC activity is required to drive these associations. In line with the idea of music as an help for cognitive functions which could lead to a deactivation of PFC activity, it has been recently shown how exposure to consonant music improve performance during a Stroop task, suggesting that music may help overcoming cognitive interference (Masataka and Perlovsky, 2013). So how could music represent a facilitatory factor particularly for words encoding? Previous EEG studies underlined how few seconds of music can influence the semantic and conceptual processes of words, showing that both music and language can prime the meaning of a word and determine physiological indices of semantic processes (Koelsch et al., 2004; Daltrozzo and Schön, 2009a,b). It is therefore possible that this semantic priming could also be reflected in easier associations and bindings between items when background music is present. Further investigations on organizational strategies during verbal encoding with music may confirm this explanation and shed new lights on music-verbal memory cognitive processes.

Another possible explanation of the music-specific PFC deactivation is an increase of attentional mechanisms in the music condition. Music is known to modulate attentional processes (Janata et al., 2002; Janata and Grafton, 2003; see also Masataka and Perlovsky, 2013) and previous fNIRS studies reported that the more attention the subjects put to a task, the more greatly rCBF (Mazoyer et al., 2002; Geday and Gjedde, 2009) and O_2_Hb concentrations (Matsuda and Hiraki, 2004, 2006) were decreased in the PFC. This second interpretation would be in line with previous behavioral studies which attributed improved cognitive performance in the presence of a musical background to higher amounts of arousal and attention (Foster and Valentine, 2001; Thompson et al., 2005; Patel, 2011). However, in apparent conflict with this interpretation, a considerable amount of literature claims the importance of the PFC in attentional processes, mainly for the maintenance and mental manipulation of memory contents (see Ptak, 2012 for a review). In the present study, we observed that PFC O_2_Hb increase seems to precede the word encoding phase by a few seconds in the most lateral fNIRS channels (i.e., left channels 1 and 2, corresponding to EEG channels AF7 and F3, **Figure 3**), and this, even in the silence condition (**Figure 3**). This may indicate that attentional processes are already in full use when encoding in silence, and put a limit on the potential of even further recruitment of attention specific to music.

Finally, we should also note that it has been repeatedly observed that music-related processing typically recruits more widely distributed networks of cortical and subcortical areas than non-musical verbal function (Halpern, 2001; Parsons, 2001; Peretz, 2002; Altenmüller, 2003; Patel, 2011). If so, PFC deactivation in the music condition could also reflect broader network recruitment during word encoding with music. Further research is needed to test this hypothesis.

fNIRS data interpretation of the present study must be done bearing in mind certain limitations. First, recent studies suggest that caution should be exercised when applying fNIRS to infer PFC activation: the task-evoked changes occurring in forehead skin perfusion could represent an overestimation of the cortical changes as measured by fNIRS. Recent reports have raised a question against the assumption that PFC O_2_Hb/HHb changes originated only from the cortical hemodynamic response (Kohno et al., 2007; Gagnon et al., 2011, 2012; Takahashi et al., 2011; Kirilina et al., 2012). Furthermore, as previously described, fNIRS acquisitions in the present study were limited to eight channels covering the bilateral DLPFC. So, it is not possible to know whether other cortical areas were involved during episodic encoding, especially for the music condition. Despite these limitations, several studies have shown fNIRS feasibility for the study of cognitive processes (Cutini et al., 2012), and this study for the first time applied fNIRS to investigate if and how music can help memory during episodic encoding.

An important perspective for further research is to apply fNIRS monitoring during the retrieval phase. Indeed, research on episodic memory during the past century has demonstrated that a complete understanding of how memories are formed requires appreciation of the many cognitive and neurobiological processes that constitute encoding and retrieval, as well as the interaction among these two stages (Brown and Craik, 2000). The behavioral and fNIRS data we obtained lead us to wonder about what is also happening during retrieval. Further studies with multichannel fNIRS systems during both encoding and retrieval phases are needed to determine which regions are more activated and to clarify how music could act on long term memory processes.

Another interesting perspective for further studies is to extend our paradigm to applications in older adults or patients with dementia. Several studies have highlighted that memory impairments in normal aging as well as several types of dementia (e.g., Alzheimer’s disease) are often linked to impairments or damage in frontal lobe functions (e.g., see Maillet and Rajah, 2012 for a review). Our results suggest that music helps verbal encoding by facilitating associative and organizational processes (i.e., generate inter-item and item-source relationships) without demanding the high-cognitive PFC processes which are usually required. At the same time, fNIRS is a non-invasive technique and its features allow it to be used also with special populations by preserving good ecological settings (Cutini et al., 2012; Ferreri et al., in press). Further fNIRS investigations on normal and pathological aging could therefore be pivotal for better understanding of how music can be used as a tool in rehabilitation of memory disorders.

In conclusion, we have shown that background music context during the encoding of verbal material modulates the activation of the PFC during encoding and, at the same time, facilitates the retrieval of the encoded material. This opens interesting perspectives on how music could act on the PFC of subjects with memory disorders for whom the prefrontal lobe is hypo-activated, impaired or damaged, such as older adults or Alzheimer’s patients.

## Conflict of Interest Statement

The authors declare that the research was conducted in the absence of any commercial or financial relationships that could be construed as a potential conflict of interest.
